# Programable Active Fixator System for Systematic In Vivo Investigation of Bone Healing Processes

**DOI:** 10.3390/s21010017

**Published:** 2020-12-22

**Authors:** Jan Barcik, Manuela Ernst, Constantin E. Dlaska, Ludmil Drenchev, Stephan Zeiter, Devakara R. Epari, Markus Windolf

**Affiliations:** 1 AO Research Institute Davos, Clavadelerstrasse 8, 7270 Davos, Switzerland; manuela.ernst@aofoundation.org (M.E.); stephan.zeiter@aofoundation.org (S.Z.); markus.windolf@aofoundation.org (M.W.); 2 Bulgarian Academy of Sciences, Institute of Metal Science ‘Acad. A. Balevski’, Shipchenski prohod 67, 1574 Sofia, Bulgaria; ljudmil.d@ims.bas.bg; 3 Orthopaedic Research Institute of Queensland, 7 Turner Street, Townsville, QLD 4812, Australia; constantin.dlaska@gmx.at; 4 Institute of Health and Biomedical Innovation, Queensland University of Technology, George Street 2, Brisbane City, QLD 4000, Australia; d.epari@qut.edu.au

**Keywords:** motorized fixation, mechanobiology of fracture healing, healing monitoring, rehabilitation, surgery

## Abstract

This manuscript introduces a programable active bone fixator system that enables systematic investigation of bone healing processes in a sheep animal model. In contrast to previous systems, this solution combines the ability to precisely control the mechanical conditions acting within a fracture with continuous monitoring of the healing progression and autonomous operation of the system throughout the experiment. The active fixator system was implemented on a double osteotomy model that shields the experimental fracture from the influence of the animal’s functional loading. A force sensor was integrated into the fixator to continuously measure stiffness of the repair tissue as an indicator for healing progression. A dedicated control unit was developed that allows programing of different loading protocols which are later executed autonomously by the active fixator. To verify the feasibility of the system, it was implanted in two sheep with different loading protocols, mimicking immediate and delayed weight-bearing, respectively. The implanted devices operated according to the programmed protocols and delivered seamless data over the whole course of the experiment. The in vivo trial confirmed the feasibility of the system. Hence, it can be applied in further preclinical studies to better understand the influence of mechanical conditions on fracture healing.

## 1. Introduction

Despite decades of research on the mechanobiology of bone repair and the development of new orthopedic implants and procedures, 5% to 10% of fractures still fail to heal properly, leading to non or delayed union [1]. Given the high incidence of fractures, the burden from healing delays is considerable—in the USA alone, up to 560,000 patients suffer from fracture healing complications each year [2].

The vast majority of bone fractures heal via indirect bone healing by development of an external callus that overgrows the fracture gap to provide stabilization and later remodels into bone [3]. It is long established that development of the fracture callus is mechanically stimulated by interfragmentary motion (IFM) in the fracture gap [4]. Effects on fracture healing have been reported for many parameters, such as amplitude of IFM [5,6], loading mode [7,8] and number of loading cycles [9,10]. However, the impact of temporal distribution of mechanical stimulus on the fracture healing process is only poorly understood. Claes et al. [11] reported that early dynamization does not improve fracture healing in a rat model and Augat et al. [12] claimed that early, full weight-bearing with a flexible external fixator delays healing in sheep. In contrast, Tufecki et al. [13] demonstrated in an ovine model that early mechanical stimulation alone permits timely bone healing and Goodship et al. [14] reported that delayed stimulation with a high strain rate inhibits fracture healing. Hence, the impact of temporal modulation of mechanical stimuli on bone healing should be investigated further, because it directly relates to the clinical rehabilitation of fracture patients.

The effects of mechanical stimulation on fracture healing have been investigated preclinically in animal models using passive or active fixators. With passive fixators [6,15,16], interfragmentary motion at the fracture gap is highly reliant on the animal’s activity and weight-bearing behavior and, hence, is only poorly controlled. In contrast, active fixators [4,5,7,9,10,13,17] are equipped with an actuator that introduces a defined loading protocol on the fracture gap. However, in most of the models the IFM in the fracture was still influenced by functional weight-bearing of the animals. Therefore, the number of loading events and the magnitude of IFM was not fully controlled.

Furthermore, in several previous studies mechanical stimulation was applied only five days per week [14,17,18], or was often not distributed over a day but applied in a single daily session [4,5,13]. Such protocols, hence, did not reflect realistic clinical scenarios.

Finally, commonly used imaging techniques (X-Ray, Computer Tomography) for assessing healing progression encounter their limits when targeting continuous monitoring of fracture healing, because they render only non-quantitative data in a poor temporal resolution [19].

In response to the limitations of radiographic evaluation, several studies have developed methods of healing assessment that investigate the progression of the mechanical properties of fracture repair tissue [20,21,22]. Typically, a sensor is attached to the fixator hardware to measure the deflection of the fixation under physiological load. The extent of the implant deflection can be corelated with the IFM in the fracture gap [23] and, to some degree, with fracture tissue stiffness [24]. Other studies suggested the application of a load cell mounted on the external fixator to measure the tissue stiffness against compression [25] or bending [26], or to measure ground reaction force and displacement of the implant with a known stiffness [27]. Except for fracture healing models, several studies have developed fixators to measure the course of healing in other bone regeneration processes, like distraction osteogenesis [28,29,30,31] or non-fracture callus induction [32].

The problem of precise control of the IFM in the experimental fracture was solved by the double osteotomy model introduced by Tufekci et al. [13]. It requires creation of two parallel osteotomies in a sheep tibia, namely a critical-size defect and an experimental defect, where healing is mechanically stimulated and monitored. The critical-size defect and the experimental defect are separated by a mobile bone fragment. The distal and proximal part of the tibia are fixed with a standard unilateral external fixator, and the mobile fragment is connected to the proximal part with an active fixator. The active fixator is equipped with an actuator that axially compresses the tissue in the fracture. This configuration shields the experimental defect from the loading that arises from the sheep’s functional weight-bearing. Hence, the IFM in the experimental defect are solely dependent on the motion of the active fixator. Yet this system did not provide a reliable measure of bone regeneration, except for conventional radiography, and the control system did not allow for a distribution of cycles that would resemble clinical scenarios

The aim of this study was to develop a system, based on Tufekci’s double osteotomy model, that enables systematic investigations of the influence of temporal variations of mechanical stimulus on fracture healing in the in vivo sheep experiment. The developed device should permit free programing of different stimulation protocols and their autonomous execution during the animal study. It should provide continuous measurement of the repair tissue stiffness as an indicator of healing progression. Eventually, the feasibility of the system should be verified in a pilot in vivo experiment.

Whereas many studies have developed tools to monitor healing progression, we aimed to combine the ability to precisely define stimulation with the continuous monitoring of tissue stiffness. Using this system, we should be able to track stiffness progression in relation to different stimulation protocols and; therefore, assess their clinical efficacy. Current clinical after-treatment protocols of fracture patients lack clear base line data and scientific evidence [33]. Generation of such data essentially requires the ability to systematically execute defined stimulation protocols with simultaneous monitoring of the healing response.

## 2. Materials and Methods

### 2.1. Fixator Setup

We employed the double osteotomy model with the active fixator described by Tufekci et al. [13] due to its unique capability of precisely controlling the IFM in the experimental defect. The model was stabilized with a conventional unilateral external fixator (DePuy Synthes, Zuchwil, Switzerland) and a custom-made active fixator. The active fixator was actuated by a Brushless Direct Current (BLDC) motor connected to a spindle drive (motor: EC32, spindle drive: GP32S, encoder: MR 512IMP, Maxon Motor AG, Sachseln, Switzerland) that moved the mobile fragment in the bone axis, thereby compressing the tissue in the experimental defect. The actual displacement of the mobile fragment was measured by the encoder embedded in the motor.

**Figure 1:**
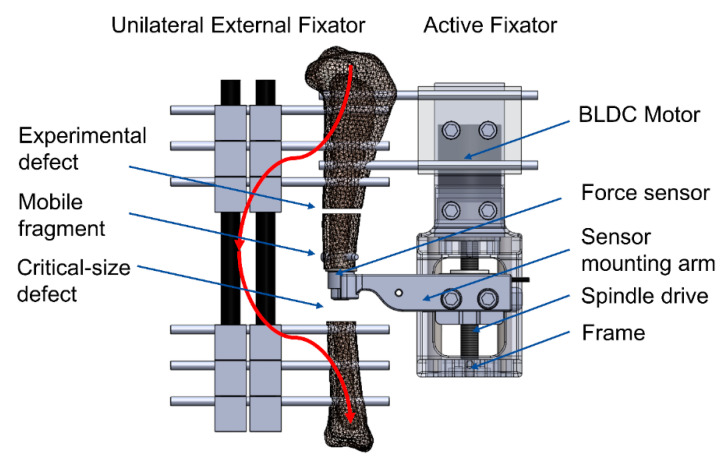
Configuration of the fracture model. The unilateral external fixator transfers forces from physiological weight-bearing from the proximal to the distal bone fragments (red line), while the experimental defect remains unaffected. The Brushless Direct Current (BLDC) motor actuates the spindle drive, which in turn moves the mobile bone fragment along the longitudinal bone axis.

In order to continuously monitor healing progression, we integrated a force sensor into the active fixator. It measures the resistance of the fracture repair tissue against compression. The sensor (S402 with additional sealing, Strain Measurement Devices Ltd., Bury St Edmunds, UK) was connected to the mobile bone fragment via a custom-made intramedullary pin fixed with an angular stable locking screw and sleeve (DePuy Synthes, Zuchwil, Switzerland). A custom-made sensor mounting arm attached the force sensor to the spindle drive (Figure 1).

The in vivo fracture stiffness was calculated as a quotient of the amplitude of the force (measured by the force sensor connected to the mobile fragment) and the displacement amplitude (measured by the encoder on the motor)—Equation (1)—where *k* is the fracture stiffness, ∆*F* is the force amplitude and ∆*d* is the displacement amplitude. Therefore, the measurement of in vivo fracture stiffness could only be calculated during stimulation periods.

(1) $$k=\frac{\Delta F}{\Delta d}$$

### 2.2. Control Unit

**Figure 2:**
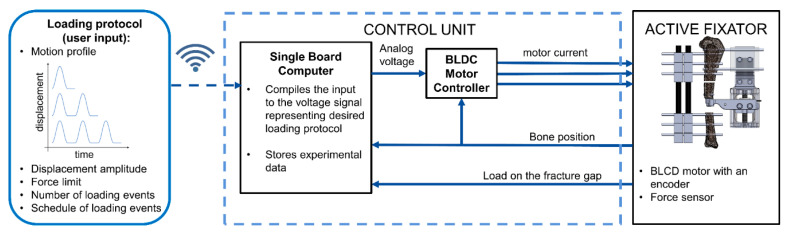
The principle of operation and flow of signals in the control unit.

A dedicated control unit directly attachable to the active fixator was developed for autonomous execution of stimulation protocols in vivo. The parameters of a particular loading protocol (motion profile, displacement amplitude, force limit (the maximum force that the tissue in the fracture gap will be subjected to during stimulation), number of loading cycles, timing and duration of loading cycles) are fed to the control unit via a C++ script that is wirelessly accessible from an external computer through the Secure Shell Protocol (SSH). The control unit then converts the stimulation parameters to an analog voltage signal, which is sent as a reference for the commanded position to the motor digital positioning controller. The controller finally adjusts the current on the motor windings to achieve the desired displacement of the spindle drive (Figure 2).

**Figure 3:**
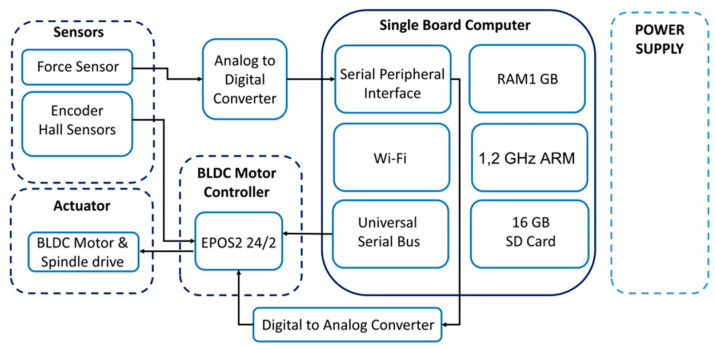
The hardware architecture of the control unit.

The control unit is based on a Single Board Computer (SBC) Raspberry Pi3 (Model B, Raspberry Pi Foundation, Cambridge, UK). Its operating system is installed on an industrial standard microSD card (S-46u, 16 GB, Swissbit, Switzerland) using a pseudo-single-level cell technology to increase the reliability of the device. Figure 3 depicts the architecture of the control unit. A digital positioning controller (EPOS2 24/2, Maxon Motor AG, Sachseln, Switzerland) operates as a slave to a master in the SBC and is configured to operate in position control mode, which adjusts the angular displacement of the motor according to the control voltage on the analog input of the EPOS2 controller. This voltage signal is generated on the SBC and sent to EPOS2 via a Digital-to-Analog Converter (MCP4822, Micro-chip Technology Inc, AB Electronics UK, Swanage, UK).

The force sensor output is converted to the digital domain (ADS1120, Texas Instruments, Dallas, TX, USA) and transferred to the SBC using a Serial Peripheral Interface protocol (SPI). The SBC is powered from the Step-Down Voltage Regulator (D24V60F5, Pololu Corporation, Las Vegas, NV, USA) and the whole system is powered by a 24 V, 60 W encapsulated power module (TMP 60124, TRACO Electronic AG, Baar, Switzerland), that can be installed at a suitable place several meters away from the control unit (i.e., at a safe distance from the animal). The device is encapsulated in a 3D-printed housing that protects the controller from mechanical stresses and contamination.

The software architecture of the control unit is based on two interconnected programs. The first code initiates the communication between the SBC and the EPOS2 controller and configures the EPOS2 controller to operate in the analog positioning mode. The latter is calculating the value of the analog control voltage for each time point during the stimulation, representing the desired position signal to the EPOS2 controller. The control voltage is programmed according to Equation (2).

(2) $$u\left(t,F\right)=\{\begin{array}{c} A\left(sin\left(2tf\pi +1.5\pi \right)+1\right)\;if\;F\leq \;F_{max}\\ u\left(t_{n-1},\;\right)\;if\;F\;>\;F_{max} \end{array}$$

where u(t,F) is the analog control voltage, t is the current time, f is the motion profile frequency, A is the stimulation amplitude, F is the current force in the fracture gap and $F_{max}$ is the force limit provided by the user. According to this formula, loading is conducted with a sinusoidal motion profile unless the force limit at the fracture gap is exceeded.

The two programs are linked with an environmental variable and the second code is executed after the first program finishes the configuration of the controller. A shell script orchestrates the execution of the programs and is run autonomously by the Linux scheduling utility ‘‘at’’ [29] according to the programmed timing of loading cycles.

### 2.3. In Vitro Testing

A series of dynamic tests was performed in vitro to determine the possible operating range of the system in terms of amplitude and frequency of stimulation. The actuator component of the system was designed by Tufeki et al. [13] however, by redesigning the control system of the actuator, the function needed revalidation. The device was instrumented on a polyurethane bone model with an empty defect, corresponding to the initial stage of healing, when the fracture gap is only filled with a hematoma [34]. A total of 9 different loading protocols consisting of 10 sinusoidal ramps with loading amplitudes from 0.1 to 1.5 mm, and loading frequencies from 0.5 to 2.0 Hz, were programmed and executed. These ranges were chosen to mimic the physiological amplitudes of IFM [35,36] and slow stepping frequencies [37]. We evaluated the deviation between actual vs. commanded peak amplitude, and actual vs. commanded frequency. The actual peaks on the displacement signal were detected as local maxima on the displacement signal.

To verify how accurately the displacement of the motor shaft corresponds to the actual displacement of the mobile fragment, the axial displacement of the mobile fragment was additionally measured with an external displacement transducer (WI, Hottinger Baldwin Messtechnik GmbH, Darmstadt, Germany). Using the previously described test setup, ten subsequent loading cycles were executed with an amplitude of 0.3 mm at 1.0 Hz. The protocol with the amplitude 0.3 mm and duration 1.0 s per stimulation cycle was further applied in the animal study.

**Figure 4:**
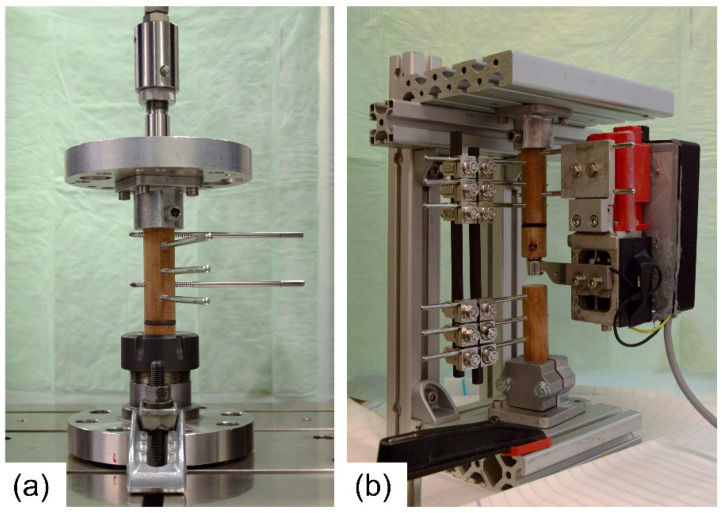
(**a**) Test setup in the universal testing machine with foam spacer (black) in the simulated experimental defect to determine actual stiffness values; (**b**) test setup with the active fixator.

To correct the stiffness measurements provided by the active fixator for influences of fixator compliance, five spacers with varying stiffness were used to simulate early stages of healing. The samples were made of foam rubbers and had a height of 3 mm and a diameter of 25 mm. Samples were placed in between two Canevasit rods (Amsler and Frey AG, Schinznach-Dorf, Switzerland, diameter 25 mm) and were loaded by a universal testing machine (5866, Instron, Norwood, MA, USA) to determine the respective defect stiffnesses (Figure 4a). With 10 N preload, 100 compression cycles were executed in displacement control at a 0.3 mm amplitude. Stiffness was evaluated from the last 10 cycles, as the average quotient of load and displacement amplitudes. Subsequently, an active fixator was connected to the Canevasit rods and the same loading and evaluation protocol was executed (Figure 4b). Here, force and displacement were recorded from the fixator’s sensors. The relation between the active fixator stiffness and the actual stiffness values was determined by a fourth-order polynomial fit (MS Excel, Microsoft, Redmond, WA, USA). The order of the polynomial was selected as the lowest order yielding a coefficient of determination R^2^ greater than 0.999. The error of the predicted stiffness was calculated for all sample points.

A submersion test of the force sensor was carried out in order to confirm its resistance to physiological fluids. The sensor was sealed with biocompatible silicone adhesive and submersed in water for 12 weeks.

### 2.4. In Vivo Application

Two sets of the active fixation system were prepared for an in vivo experiment. The sensors were sealed with biocompatible adhesive and were subsequently calibrated using a material testing machine for axial compression in the range from 0 N to 600 N (5866, Instron, Norwood, MA, USA). The calibration was performed in a water bath at 39 °C, corresponding to the body temperature of a sheep [38]. Afterwards, each sensor was assembled with a sensor arm and an intramedullary pin. The sensors and the fixators were sterilized using ethylene oxide.

**Figure 5:**
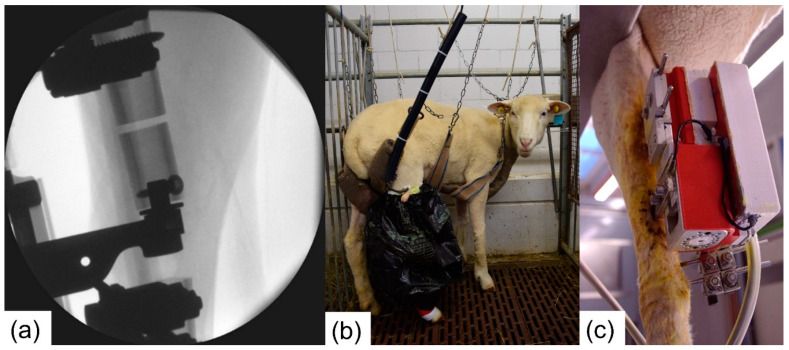
(**a**) Intraoperative radiograph showing the attachment of the force sensor on the mobile fragment and the 3.0 mm experimental defect. (**b**) Postoperative housing of the animals in the sling system. (**c**) Active fixator and attached control unit in the in vivo trial.

Two female Swiss White Alpine sheep were procured under the approval of the ethics committee of the canton of Grisons in Switzerland (Ethics committee approval number TVB 2017_23). Under general anesthesia and appropriate analgesia, the fixators were placed on the right tibia of the sheep. The procedure started by positioning the unilateral fixator on the medial aspect of the tibia. Subsequently, the active fixator pins were placed on the anterior aspect. A 3.0 cm wide critical-size defect was then created using an oscillating saw and a cutting guide. The tibialis cranialis muscle was split to insert the pre-assembled sensor arm with the force sensor and intramedullary pin. Next, the frame of the active fixator was placed between the active fixator pins and the sensor arm. The sensor was fixed subsequently to the proximal fragment of the osteotomized tibia using an angular stable screw interlocking with the custom-made intramedullary pin. Using an oscillating saw and the cutting guide, the experimental defect was created. The spindle of the active fixator was manually rotated to move the mobile fragment down until a gap width of 3.0 mm was obtained (Figure 5a). The gap width was verified with a 3.0 mm spacer. All cutting and drilling procedures were conducted under irrigation with ringer solution to prevent excessive heat generation. After closure and bandaging of the surgical site, the control unit was attached to the motor and the fixation frame (Figure 5c).

Post-surgery, the sheep were supported in a sling system, but not fully immobilized, for the entire study duration (Figure 5b). The sling system allows the sheep to fully bear weight but prevents excessive peak loading on the osteotomies when the animals lie down or stand up. Moreover, the animals are able to lie down in the harness. Similar systems have been reported for fracture healing experiments in sheep [39].

To minimize the risk of infection, regular pin cleaning was performed under sedation. Radiographs were taken on a weekly basis in a plane inclined 45° from the mediolateral plane towards the cranial direction. The callus area was measured manually on each radiograph (Synedra View Personal, Synedra Information Technologies GmbH, Innsbruck, Austria).

Each sheep was subjected to a different stimulation protocol. The system was configured to mimic immediate weight-bearing for one sheep (stimulation from the day 1 post-op) and delayed weight-bearing (stimulation from the day 22 post-op) for the second one. During the stimulation period, the motor executed 1000 loading cycles per day with an amplitude equal 0.3 mm. The amplitude and the number of loading cycles were selected based on the literature [9,16]. The loading cycles were equally distributed over 12 h (9 a.m.–9 p.m.). The cut of force was set to 300 N.

The animals were euthanized nine weeks post-surgery with an overdose of pentobarbital. Both the operated and the contralateral limbs were dissected, and the fixators were removed from the bones. For subsequent histological analysis, the samples were fixed in 70% ethanol, dehydrated and embedded in resin. Afterwards, the samples were sliced into 100 µm thick sections and stained with Giemsa-Eosin. A descriptive qualitative analysis of the stained sections was conducted by a certified veterinary pathologist to investigate the stage of bone healing and identify potential signs of necrosis.

To verify unaltered performance of the force sensors, the explanted sensors were subjected to the same loading protocol that was applied during the pre-op calibration procedure again.

All active fixator measurements were retrospectively converted according to the described in vitro testing procedure.

## 3. Results

### 3.1. In Vitro Testing

In all protocols that were tested during the dynamic in vitro tests, the stimulation frequency corresponded well to the commanded frequency with deviations below 3%. For the low-frequency protocols (0.5 and 1.0 Hz), the actual peak amplitude replicated properly the commanded one across all tested displacement amplitudes, whereas at 2.0 Hz the deviation from the commanded displacement exceeded 5% at 1.0 and 1.5 mm (Table 1).

The second in vitro test—that examined the protocol used in subsequent animal study—revealed that the displacement of the motor shaft corresponds accurately to the actual displacement of the mobile fragment, with an average deviation of 2.75 ± 0.06% of the commanded displacement.

**Figure 6:**
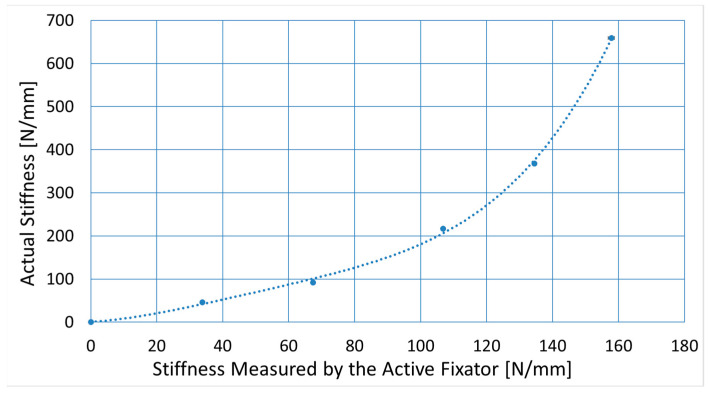
The relation between the stiffness of the experimental defect measured by the active fixator and the actual stiffness measured by a universal testing machine. Each point represents the average of 10 loading cycles. All standard deviations were lower than 2 N/mm. The coefficient of determination (R2) was > 0.999.

Figure 6 depicts the results of the stiffness correction procedure. The fourth-order polynomial curve (R^2^ > 0.999) estimates the relation between the actual stiffness (y) and the stiffness that was measured by the active fixator (x). Respectively, Equation (3) was used for data conversion. The maximum error between the fitting curve and the true stiffness was 10.96 N/mm for the sample with the actual stiffness value of 217 N/mm.

(3) $$y\;=\;0.00000257x^{4}\;-\;0.00049205x^{3}\;+\;0.03728401x^{2}\;+\;0.42061693x\;+\;0.90770743\;$$

The submersion test confirmed the resistance of the force sensor to physiological fluids. The sensor maintained its function throughout the submersion experiment.

### 3.2. In Vivo Application

Both surgeries were performed without complications. An accurate transverse experimental defect was created and precise alignment of the mobile segment and proximal bone fragment was achieved (Figure 5a). The animals tolerated the external fixators and the applied stimulation protocol well and did not show any problem during the recovery until they were euthanized nine weeks post-op.

**Figure 7:**
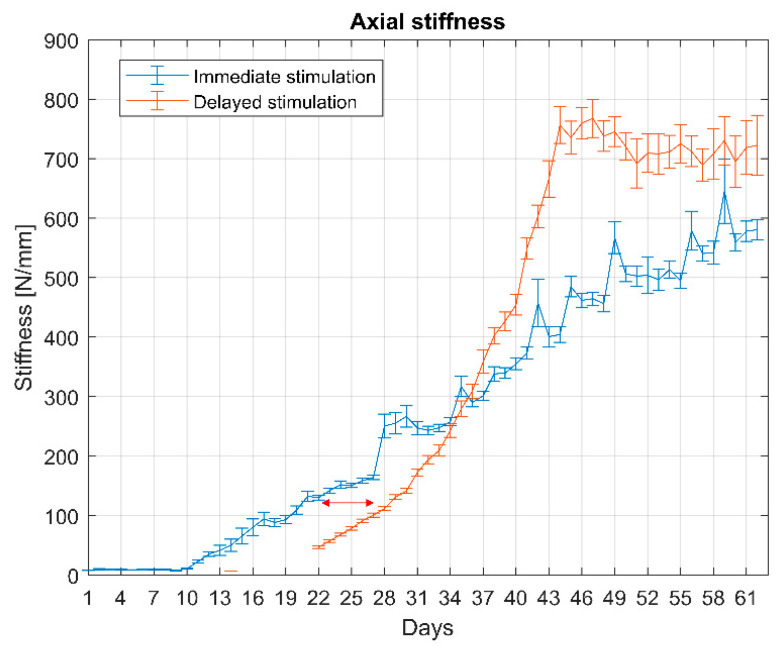
Measurements of the in vivo stiffness show that the delayed stimulated fracture consolidates approximately one week later as compared to the immediately stimulated (arrow). The daily results are presented as the average of all 1000 daily measurements and the bars represent standard deviation. An additional measurement (10 loading cycles) was performed on the animal with delayed stimulation on day 14 to acquire a baseline value of the fracture stiffness.

Automated measurements of the resistance of the repair tissue enabled continuous monitoring of the in vivo fracture stiffness throughout the experiment. The fracture stiffness (Figure 7) in the animal with the immediate stimulation began to increase from the tenth postoperative day, while, in the second animal, the stiffening of the fracture was delayed for seven days. The stiffness plateaued between the sixth and seventh week after the surgery for the delayed stimulation animal.

**Figure 8:**
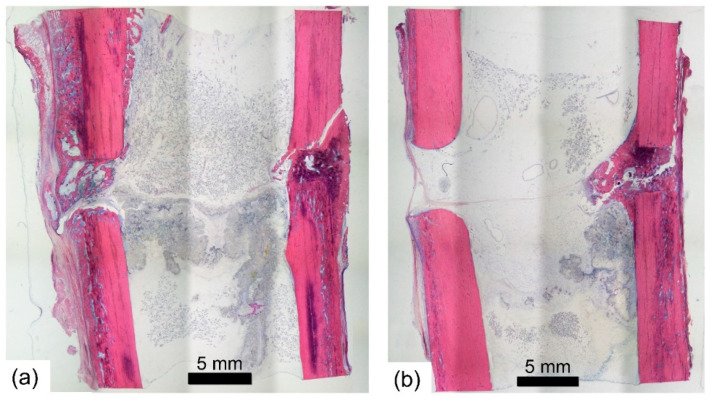
Giemsa-eosin-stained sections showing the experimental defects of both animals (immediate (**a**) and delayed (**b**) stimulation). In both animals the isolated mobile fragment was vital.

The maximum callus area measured on radiographs for the immediately stimulated animal was equal to 36.8 mm^2^ and for the delayed on 3.57 mm^2^. The histological analysis of the samples (Figure 8) did not reveal any signs of bone necrosis in case of both animals, but partially showed high remodeling of the tissue in the fracture gap.

The post-mortem testing of the force sensors used in vivo did not reveal any changes with respect to the calibration conducted prior to the experiment—differences in the slope of the calibration curve were smaller than 1%.

## 4. Discussion

We developed a system to investigate the influence of temporal variations of mechanical stimulus on fracture healing in vivo. Our system is based on a previous model described by Tufekci et al. [13]; however, we have extended the capability of this system by enabling continuous monitoring of fracture in vivo stiffness due to the integration of a force sensor. Moreover, an electronic control unit that autonomously executed the stimulation protocols and collected experimental data were developed. The control system described in this manuscript is now able to execute a vast variety of complex protocols with different distributions of loading cycles with variable stimulation amplitudes and frequencies and thus mimic different rehabilitation scenarios.

The system was first tested in vitro and was implanted in two sheep for a period of nine weeks subsequently. The in vitro tests determined the operating range of the active fixator in terms of stimulation frequency and amplitude and demonstrated the robust operation of the system, thereby qualifying it for in vivo application. During the in vivo trial, the implanted devices maintained their functionality over the whole course of the experiment (i.e., the system executed stimulation according to the programmed protocols (immediate and delay stimulation) and continuously measured the stiffness of the fracture repair tissue).

In this feasibility study we simulated a simplified clinical daily scenario with 12 h of activity followed by 12 h of rest. The configuration of the double osteotomy model guaranteed that the IFM in the fracture gap were purely controlled by the external active fixator and that no stimulation was applied during resting. Hence, the model is capable of precise simulation of different postoperative stimulation protocols in vivo and, thanks to autonomous operation, it can mimic more clinically relevant stimulation protocols than most previous studies.

The integration of the force sensor into Tufekci’s active fixator allowed to continuously monitor the repair tissue stiffness as an indicator of healing progression. Previously, Bishop et al. [25] described an active fixation system that measured the in vivo compression stiffness in sheep, but the force sensor was mounted externally on the active fixator. In this arrangement, the force does not describe the pure callus response because the sensor is mounted off axis the fractured bone. In contrast, we mounted the load cell in line with the bone axis.

Whereas many studies described devices that can measure healing progression, to the best of our knowledge, our system is the first one that precisely defines the mechanical stimulation protocol in a fracture healing model—ensuring that no stimulation is performed during resting—and simultaneously measures the fracture stiffness. The system that we have developed operates autonomously; however, the model is labor-intensive in terms of surgical complexity and post-operative care efforts.

During the in vivo trial, the fracture stiffness started to increase around day 10 post-op. The measured values are of the magnitude as previously reported by Bishop [40] and Goodship et al. [17] until four weeks post-op. In the early phase of healing, the model is; therefore, deemed to deliver a reliable estimation of repair tissue stiffness. However, in the later stage the values saturated at lower stiffness levels than in previous studies. We assume that the repair tissue was not fully compressed anymore by the actuator at that stage, but instead, due to the compliance of the system, part of the actuator motion resulted in bending the pins rather than compressing the tissue.

Consequently, we converted the in vivo stiffness measurements according to the results from the in vitro test, where the fixator was mounted on a rod mimicking cortical bone. However, a straight rod is only an approximation of the in vivo situation and does not account for the influence of tibia shape and the presence of soft tissue. Moreover, the test has shown that a reliable approximation is only valid in the early phase of tissue stiffening. At later maturation (from the sixth to the ninth week), a plateau in the in vivo stiffness curve becomes apparent. Thus, the model targets early phases of callus healing before remodeling processes start to dominate. Consequently, the stiffnesses of the surrogate materials used to simulate healing stages during in vitro testing were selected to match the stiffness window relevant for the model.

The impact of the structural compliance of an external active fixator system has been previously reported [41], but cannot be entirely eliminated from such a model due to the generally long moment arms of external fixators. It could be reduced though, for example, by using pins with a larger diameter or by positioning the fixator as close to the bone as possible. However, pins that are too large bear the risk of pin site fractures, and shorter moment arms are difficult to achieve in vivo because sufficient space should be left to enable cleaning of the external fixator pins during the experiment.

Although the fixator was explicitly designed to eliminate the effect of animal’s weight-bearing, some mechanical response of the tissue in the critical-size defect cannot be fully excluded.

The size of the callus observed in this study was considerably smaller than what has been reported for a 3.0 mm defect in sheep [42,43,44]. The small callus formation might be attributed to either an insufficient level of stimulation—because the magnitude of stimulation was swallowed partially by the fixator’s structural compliance—or to impaired perfusion at the fracture site. Although the histological analysis did not reveal any signs of osteonecrosis, fixation of the force sensor to the mobile bone segment through the intramedullary pin could have decreased its perfusion and, thereby, compromised the healing process. However, the results from a previous study utilizing a similar model (Tufekci et al. [13], 15 animals) have proven a robust healing response despite compromised vascularity. Therefore, in our opinion, the model can indeed be regarded as valid and unique approach to study the mechanics of fracture healing. Moreover, compromised vascularity is also frequently seen in clinics in open fracture cases, high energy polytrauma and segment transport.

Our model was developed with a special focus on investigating the impact of different temporal distributions of mechanical stimulation. The double osteotomy model allowed us to precisely apply stimulation and assure that no stimulation was applied during resting periods. A recent study by Hente and Perren [10] pointed out that resting time between stimulation cycles might be a crucial and underestimated parameter determining fracture healing. With “conventional” models, the differentiation between resting and stimulatory periods would not be possible due to the effect of uncontrolled weight-bearing.

The goal of this manuscript was to introduce a framework for systematic investigations of the influence of temporal variations of mechanical stimulus on fracture healing and discuss the feasibility of this system; therefore, we do not compare the healing progression results between the animals.

Future improvements of the system should focus on strengthening the structure of the active fixator, to decrease the effect of its compliance in order to prolong the time frame, whereby we could apply defined stimulation and reliably monitor the progression of fracture stiffness. In future applications of this system in vivo, each fixator should be individually tested prior to implantation to estimate the structural compliance of the fixators. Finally, the device is very invasive, and perfusion of the fracture gap should be closely monitored in subsequent studies to avoid inconclusive results because of impaired local blood supply.

## 5. Conclusions

We developed an autonomously functioning active bone fixator system, for large animal research, that allows well-controlled interfragmentary motion to be installed in the fracture gap independent of functional loading of the animal. The in vivo trial confirmed the feasibility of the applied solutions. With its capability to operate autonomously, the system will facilitate animal studies investigating the influence of temporal variations of mechanical stimulus on fracture healing, which are important for future optimization of the clinical rehabilitation protocols for fracture patients.

## Figures and Tables

**Table 1:** Results of the fixator dynamic test. AD—actual displacement; D—difference between actual and commanded displacement as percentage of commanded displacement. The results are reported as mean and standard deviation.

		Frequency [Hz]					
		0.5		1.0		2.0	
		AD [mm]	D (%)	AD [mm]	D (%)	AD [mm]	D (%)
**Commanded** **Displacement** **[mm]**	**0.1**	0.097 SD < 0.01	2.99 SD = 0.07	0.097 SD < 0.01	2.97 SD = 0.09	0.097 SD < 0.01	3.01 SD = 0.14
	**1.0**	0.993 SD < 0.01	0.71 SD = 0.02	0.994 SD < 0.01	0.65 SD = 0.01	0.941 SD < 0.01	5.918 SD = 0.5
	**1.5**	1.492 SD < 0.01	0.52 SD = 0.021	1.516 SD < 0.01	1.05 SD = 0.08	1.209 SD = 0.044	19.44 SD = 2.94
